# Surfactant Control of Coffee Ring Formation in Carbon Nanotube Suspensions

**DOI:** 10.1021/acs.langmuir.2c01691

**Published:** 2023-01-06

**Authors:** N. S. Howard, A. J. Archer, D. N. Sibley, D. J. Southee, K. G. U. Wijayantha

**Affiliations:** † Department of Chemistry, Loughborough University, Loughborough LE11 3TU, U.K.; ‡ Department of Mathematical Sciences, Loughborough University, Loughborough LE11 3TU, U.K.; § Interdisciplinary Centre for Mathematical Modelling, Loughborough University, Loughborough LE11 3TU, U.K.; ∥ School of Design and Creative Arts, Loughborough University, Loughborough LE11 3TU, U.K.; ⊥ Centre for Renewable and Low Carbon Energy, Cranfield University, Cranfield, Bedfordshire MK43 0AL, U.K.

## Abstract

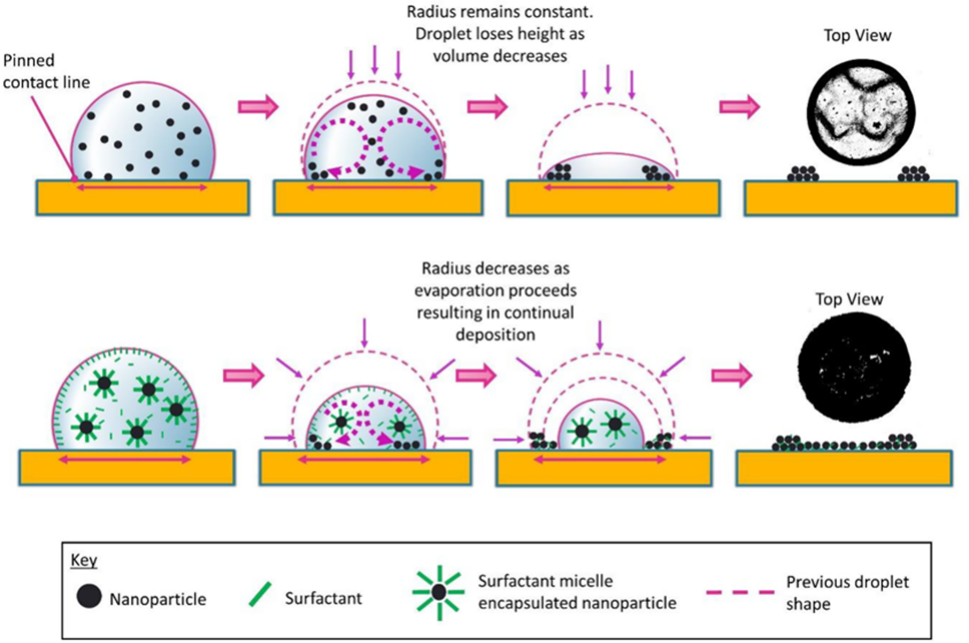

The coffee ring effect regularly occurs during the evaporation of colloidal droplets and is often undesirable. Here we show that adding a specific concentration of a surfactant can mitigate this effect. We have conducted experiments on aqueous suspensions of carbon nanotubes that were prepared with cationic surfactant dodecyltrimethylammonium bromide added at 0.2, 0.5, 1, 2, 5, and 10 times the critical micelle concentration. Colloidal droplets were deposited on candidate substrates for printed electronics with varying wetting characteristics: glass, polyethylene terephthalate, fluoroethylene propylene copolymer, and polydimethylsiloxane. Following drying, four pattern types were observed in the final deposits: dot-like, uniform, coffee ring deposits, and combined patterns (coffee ring with a dot-like central deposit). Evaporation occurred predominantly in constant contact radius mode for most pattern types, except for some cases that led to uniform deposits in which early stage receding of the contact line occurred. Image analysis and profilometry yielded deposit thicknesses, allowing us to identify a coffee ring subfeature in all uniform deposits and to infer the percentage coverage in all cases. Importantly, a critical surfactant concentration was identified for the generation of highly uniform deposits across all substrates. This concentration resulted in visually uniform deposits consisting of a coffee ring subfeature with a densely packed center, generated from two distinct evaporative phases.

## 1 Introduction

### 1.1 Self-Assembly

In the late 1990s and early 2000s, seminal work by Deegan et al. on the evaporation of droplets containing suspended particles identified radial outward flow and a pinned contact line to be the cause of the frequently observed “coffee ring effect” (CRE).^1−3^ Since this work, many distinct, ordered pattern types such as stick–slip,^4−9^ dot-like,^10−12^ and radial cracking^13−15^ have been identified and reported from the evaporation of droplets containing various suspended particles under a wide variety of experimental conditions. The emergence of such structures from complex fluids is a consequence of the interplay between many physiochemical effects. It can be influenced by solute characteristics such as size,^16−18^ shape,^19^ particle–substrate adhesion,^20^ and aspect ratio,^21^ as well as solute concentration.^22,23^ The composition of the base fluid also plays a key role, specifically characteristics such as surface tension,^24^ pH,^10^ viscosity and volatility^25^ as well as the presence of specific additives such as salts^26^ or surfactants.^27^ Drying characteristics such as evaporation rate, localized variations in solute concentrations, droplet internal flow, and variations in both contact angle and radius also have an influence.^28^ These depend on atmospheric conditions like temperature^17,29^ and humidity.^30^ Furthermore, external factors, such as the characteristics of the substrate like surface wettability,^12,29^ adhesive nature,^31^ texture,^32,33^ and viscoelastic nature^34^ are critically important. These factors and the interplay between them play a key role in influencing the thermodynamic interactions and processes that govern the mass transport that, ultimately, determines the final morphology of the solid matter. Collectively, the processes responsible for the generation of these ordered deposits from previously uniformly dispersed material are termed self-assembly.

### 1.2 Existing Applications and Challenges Associated with Emerging Applications

Considerable research has been conducted on this topic as the potential applications that stem from the ability to predict and control self-assembly are numerous and span a great number of industries.

For example, anticounterfeit uses have been identified on the basis of altering the substrate temperature and wettability, which was found, in turn, to alter the crystalline ordering in the self-assembly of particles sufficiently to produce photonic variations in inkjet-printed images.^12^ Analysis of the drying of biological fluids has shown that variations in solute type, and concentration, can create distinct deposition patterns with promising implications for rapid disease screening and diagnostics.^35^ Positive responses to treatments were also identifiable in the patterns formed from dried blood serum of patients with gastrointestinal disease.^36^ Functionalized gold nanoparticles have been shown to produce self-assembled crystalline lattice structures with enhanced light absorption in thin layers suitable for high-efficiency solar cells^37^ and other such structures with potential useful applications. The engineering of surfaces with antiviral properties^38^ and allergen detection in foods^39^ are additional examples of the many applications that stem from the self-assembly of nanoparticles within droplets.

Despite these uses, there are many instances in which self-assembly within liquid droplets proves to be detrimental and mitigating this behavior becomes a crucial objective. Examples include microarray manufacturing^40,41^ in which the CRE can affect the distribution density of microspots and result in an increased incidence of cross contamination between neighboring samples. The CRE can also prove to be a hindrance in Raman spectroscopy where the irregular distribution of particles due to the CRE can produce unreliable signals.^42^ Other examples include biochemical assays^43,44^ and infrared spectroscopy.^45^

One area where nanoparticle-laden droplets possess vast potential is inkjet printing for printed electronic applications. Due to their conductive nature, silver and carbon nanoparticle-based inks have established their place as the primary materials of focus for printed electronics applications.^46,47^ However, as inkjet methods are dependent upon droplet evaporation and the deposition of the active, conductive material, self-assembly and the CRE still present an issue in the further advancement of inkjet printing. Several methods have been proposed to mitigate this problem but often include additional preparation steps or temperature-controlled environments.

Herein, we present our recent findings on the patterns formed from colloidal droplets containing rod-shaped, conductive carbon nanotubes (CNTs) and varying concentrations of a surfactant. The deposition patterns are analyzed and classified via image analysis software, interferometry, and microscopy, and conditions for increasing deposit uniformity are identified. A critical surfactant concentration is identified for the generation of uniform CNT deposits under ambient conditions on a variety of substrates. The reason for considering this system on these surfaces is they have potential applications in printed electronics.

## 2 Experimental Section

### 2.1 Colloidal Preparation

Multiwalled carbon nanotubes (mwCNTs) with average external dimensions of 6–9 nm × 5 μm were purchased from Sigma-Aldrich. The sample purity with respect to dispersity was >95%. These were used as received, with no treatment to alter the surface chemistry. Cationic surfactant dodecyltrimethylammonium bromide (DTAB), obtained from ACROS Organics, was used to prepare aqueous surfactant stock solutions via serial dilution at concentrations of 0.2, 0.5, 1, 2, 5, and 10 times the experimentally determined critical micelle concentration (CMC), in deionized water obtained from a PURELAB DV 35 instrument fitted with an ELGA Biofilter. The desired mass of mwCNTs was weighed to the nearest 0.1 mg, and the required quantity of the surfactant stock was added. Samples were then homogenized via an IKA Ultra-Turrax T-25 Disperser probe set at a speed of 6500 rpm for 1 h. Following this, samples were left to rest for 2 h to ensure no visible foam or bubbles were present, before a final ultrasonication step to promote mwCNT dispersion and debundling, for a further 2 h.

All samples were agitated briefly via a vortex shaker set at 24 000 rpm for 30 s prior to any subsequent analysis.

Then, 0.5 ± 0.025 μL carbon nanotube (CNT)-laden aqueous droplets were deposited via micropipette on prepared substrates under ambient conditions (with an average temperature or 21.2 ± 0.6 °C and an average relative humidity of 36.2 ± 5.7%).

### 2.2 Critical Micelle Concentration Determination

Aqueous solutions of the surfactant were prepared at concentrations ranging from 0.1 to 20 times the published CMC. An optical tensiometer in pendant drop mode and OneAttension software were used to determine the surface tension of each solution at each concentration, and the results are summarized in Table 1. The volume of the droplets was 1.1 μL (±0.3), and each measurement was performed in triplicate. The CMC values were estimated at the point where the decrease in surface tension plateaus, when plotted against an increasing surfactant concentration. The values obtained are summarized in Table 1.

**Table 1:** Experimental Determination of the Critical Micelle Concentration of Dodecyltrimethylammonium Bromide in Deionized Water

	DTAB concentration at CMC (mg/mL) (±SD)		DTAB molarity at CMC (mM) (±SD)		DTAB surface tension at CMC (mN/m)	
molar mass (g/mol)	reference^48^	measured	reference	measured	reference^48^	measured
308.4	4.5	4.1 (0.09)	14.6	13.1 (0.3)	37.6	39.4

### 2.3 Substrate Preparation

The clear glass slides were obtained from Sail Brand, and polyethylene terephthalate (PET) and fluoroethylene propylene copolymer (FEP) from DuPont Teijin Films. These substrates were used as received with no other treatment except cleaning prior to use. The substrates were selected as they represented a range of hydrophobic, hydrophilic, adhesive, and non-adhesive substrates. In addition, all substrates were transparent, which enabled greater analysis of the final deposits. To clean the substrates, a method based on that of Stanton et al.^49^ was employed. The cut substrates were sonicated in ethanol for 30 min, rinsed in fresh ethanol, and dried in a stream of nitrogen. The rinsed substrates were then placed in an oven for 30 min at 70 °C to remove any residual ethanol.

Hydrophobic polydimethylsiloxane (PDMS) substrates were prepared from a Sylgard-184 kit from Sigma-Aldrich, using PET as a cast. Prior to use, the PET was cut to size and sonicated in ethanol for 30 min, rinsed in fresh ethanol, and dried in a stream of nitrogen. The PDMS prepolymer components (10:1 elastomer:hardener) were introduced to each other in a sealed packet and then manually massaged in the packet. After this, the packet was sonicated for 30 min to ensure complete mixing; then, 1 g of the combined PDMS prepolymer was poured onto each precleaned PET. The coated PET was then placed under reduced pressure, 0.1 mbar, for 1 h and then placed on a hot plate (*T* = 120 °C for 3 h) to cure. After curing, the PDMS was peeled from the cast, further sonicated in ethanol, and again dried in nitrogen.

### 2.4 Substrate Characterization

Contact angle hysteresis, adhesion, and roughness measurements were performed on each substrate.

#### 2.4.1 Contact Angle

Contact angle measurements were determined via a Theta Lite optical tensiometer in sessile droplet mode and analyzed with OneAttension software. Droplets were analyzed and recorded for 10 s once each droplet had steadied on the substrate, at a rate of 8.7 frames per second, and one data point was produced for each frame taken. All values reported are mean values from each data set. This setup was also utilized to determine evaporation mode at different times during droplet evaporation. Constant contact radius (CCR) refers to stages of evaporation where the contact line is pinned and therefore remains constant as evaporation proceeds. Conversely, constant contact angle (CCA) refers to situations in which the contact angle remains roughly fixed over time, while the contact radius simultaneously decreases as the contact line moves inward and the droplet volume decreases during the evaporation process.

Contact angle hysteresis is the difference between the advancing contact angle (θ_A_) and the receding contact angle (θ_R_), which for a particular droplet–substrate interaction, leads to a range of observable values for the apparent contact angle. θ_A_ and θ_R_ represent the maximum and minimum observable values for a specific solid and liquid phase combination before droplet depinning occurs. Beyond these values, the droplet would begin to move and become displaced from its original position.

To obtain equilibrium contact angle (θ_E_) values, measurements were taken on a horizontal stage. For θ_A_ and θ_R_, droplets were placed on an inclined stage angled 45° from the horizontal plane. A 2 μL droplet of deionized water was added to each angled substrate. θ_A_ was measured as the larger angle on the “downhill” side of the substrate, and conversely, θ_R_ was measured as the angle on the “uphill”. Two microliters of water was added to the droplet, and θ_A_ and θ_R_ were recorded for the new volume. This continued until the values plateaued or the droplets were no longer stationary. Three different samples of each substrate were analyzed, and the data from each sample were recorded in triplicate and averaged. Hysteresis was calculated for each substrate as the difference between θ_A_ and θ_R_.

#### 2.4.2 Adhesion

Adhesion was determined using a modification of a method described by Stanton et al.,^49^ determined by the maximum loading volume of deionized water on each substrate set at a 45° angle before the droplet began to move “downhill”. To start, a 2 μL droplet was deposited on the substate. As for the advancing and receding contact angle measurements, this was increased in 2 μL increments until droplet movement was observed. Once a rough volume had been determined via this method, the process was repeated this time with an initial droplet that was 5 μL smaller than the previously determined rough value. An additional volume of 1 μL was added to each droplet to determine the roll off volume to the nearest 1 μL. This method was performed on three samples of each substrate, with each sample being tested in triplicate, and the results were averaged.

#### 2.4.3 Roughness

The roughness of each substrate was determined via a white light interferometer (NPFLEX Bruker) and calculated as the root mean square (*R*_rms_) of the peak and trough heights of the test surface.

### 2.5 Deposit Analysis

ImageJ (National Institute of Health, Bethesda, MD)^50^ with an additional interactive three-dimensional (3D) surface plot tool^51^ was used for analysis via optical microscopy. All deposit profiles were analyzed via the NPFlex Brunker white light interferometer set at 50 times magnification and Vision64Map software. Additional analysis was conducted via scanning electron microscopy to determine characteristics such as particle location, packing, and density.

#### 2.5.1 Classification

Deposits were classified in accordance with the literature for common pattern types such as coffee ring and dot-like.^52^ In this research, the term “uniform” was used to describe deposition patterns that showed percentage coverage of >65%, as this was the cutoff between coffee ring patterns with light central deposition and patterns with heavier central coverage. The percentage coverage was determined using ImageJ as follows. A square region was selected directly around each circular deposit so that each side of the selected area touched the deposit outer perimeter at one point, roughly at the midpoint of each side of the square. The selected zone was used to create a histogram, plotting the pixel intensity frequency within the selected area of the image. The image pixels were mapped to 8 bit, meaning each pixel wais assigned an intensity value ranging from 0, corresponding to pure black, to 255, corresponding to pure white. As the CNT particles were black in color, areas with heavier deposition appeared darker, allowing a pixel intensity scale to act as a reasonable proxy for CNT nanoparticle density. For each image analyzed, the lowest pixel intensity observed was indeed 0, corresponding to regions of complete CNT deposition and no visible substrate. The highest pixel intensity recorded from the images varied and depended upon factors like substrate type and microscope lighting. The maximum pixel intensity values ranged from 126 to 193 in all cases. For each deposit image, this value was taken as the intensity value for the bare substrate, free from any particles as the selected analysis region always included a contribution from the area outside the initial droplet wetting area. The percentage coverage was calculated as follows:
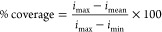
where *i*_mean_ is the mean pixel intensity, *i*_max_ is the maximum pixel intensity (corresponding to the bare substrate), and *i*_min_ is the minimum pixel intensity (corresponding to areas of greatest deposit thickness). This percentage coverage allowed us to determine the proportion of the selected region that had a pixel intensity lower than the mean, corresponding to high nanoparticle coverage. This value was used as an estimate of the degree of coverage to represent deposit percentage uniformity.

## 3 Results and Discussion

Presented here are the results derived from the evaporation of 0.5 ± 0.025 μL carbon nanotube (CNT)-laden aqueous droplets, deposited under ambient conditions (with an average temperature of 21.2 ± 0.6 °C and an average relative humidity of 36.2 ± 5.7%), on a range of four different substrates. Each substrate was characterized in terms of properties such as wettability, water adhesion, and roughness as described in the previous section, and each suspension–substrate combination was tested in triplicate. The samples contained cationic surfactant DTAB, prepared at concentrations ranging from 0.2 to 10 times the experimentally determined CMC (equivalent to 1.5–146 mM), selected to span a wide range of values to enable analysis of subsequent surfactant-mediated behavioral changes. The mwCNT nanoparticle loading was 3.9 mg/mL. The droplets were tracked throughout the entire evaporation process for each formulation, and the subsequent deposits were analyzed to determine the final morphology.

### 3.1 Substrate Characterization

All substrates used were subjected to characterization prior to any droplet deposition. The results are presented in Table 2 and include the aqueous equilibrium contact angle (θ_E_), aqueous roll off volume, hysteresis, roughness, thickness, and surface energy.

**Table 2:** Average Characteristic Properties of the Four Substrates Determined Using Water Droplets^a^

substrate	θ_E_ (deg) (±SD)	θ_A_ (deg) (±SD)	Θ_R_ (deg) (±SD)	hysteresis (deg) (θ_A_ – θ_R_)	roll off volume (μL)	roughness (nm) (*R*_rms_)	thickness (μm) (±SD)	surface energy (nM/m)
glass	59 (0.2)	65 (0.3)	37 (0.2)	28	14	3	108.1 (0.2)	70^53^
PET	77 (0.9)	97 (1.0)	54 (1.5)	43	18	16	196.7 (4.2)	42^54^
FEP	107 (1.9)	111 (2.5)	69 (0.6)	42	12	22	106.7 (2.1)	20^55^
PDMS	113 (1.9)	116 (1.5)	55 (0.6)	61	40	5	383.0 (73.9)	20^55^

a These include average advancing and receding contact angles (θ_A_ and θ_R_, respectively) to the nearest degree and roll off volume with the substrate at a 45° angle to the nearest whole microliter. For all contact angles, the measured standard deviation was <2°. Note, however, that the range from θ_A_ to θ_R_ gives the plausible range of equilibrium contact angle values; i.e., these define an effective error range. Surface energy values were obtained from the literature with reference numbers shown in parentheses.

### 3.2 Deposition Patterns

**Figure 1:**
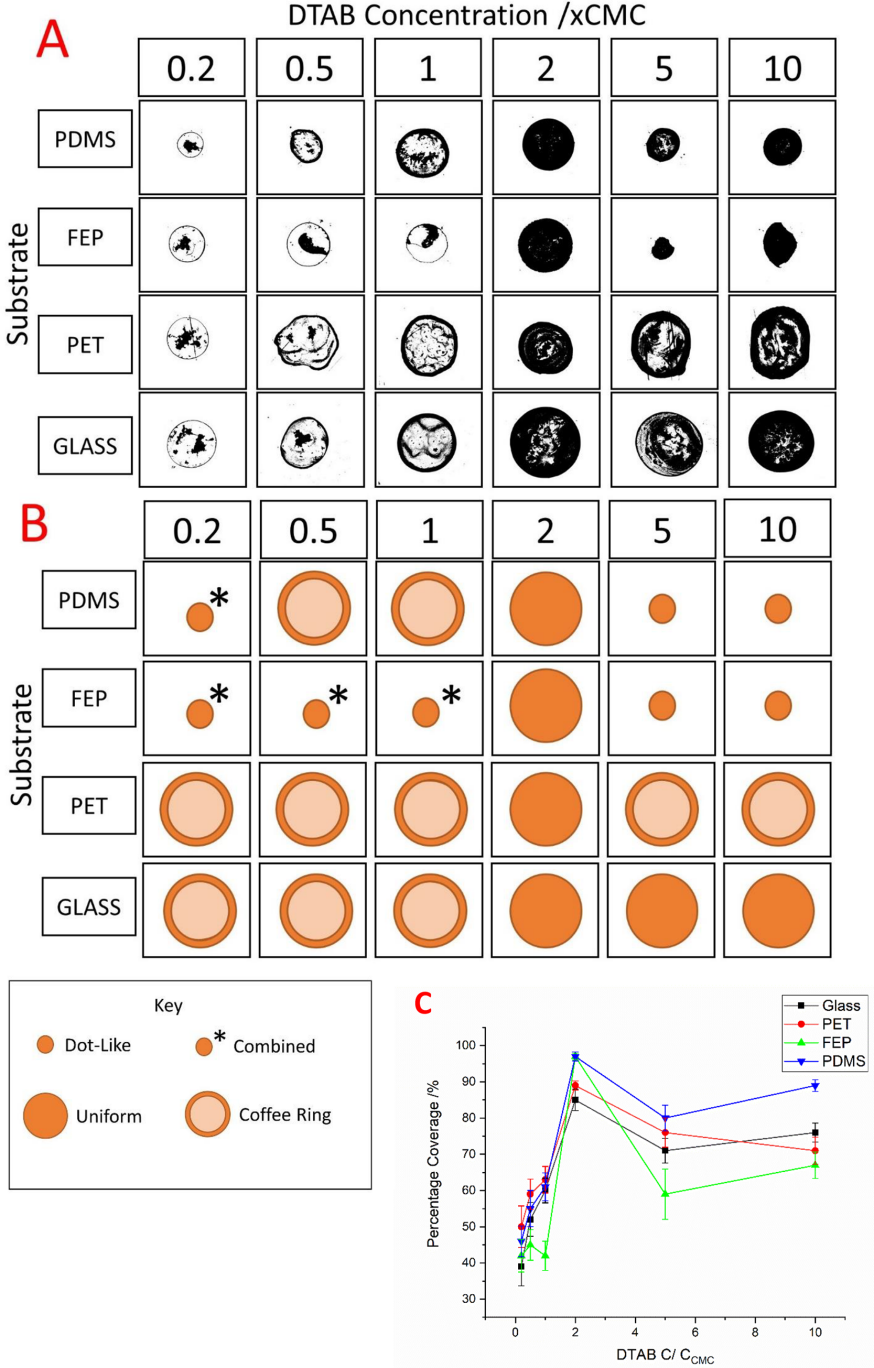
(A) Overhead view of dried deposits on various substrates. (B) Schematic representation of deposit classification. (C) Average deposit uniformity determined by percentage coverage plotted vs DTAB concentration as a ratio of the CMC.

In this research, four deposit profiles were observed: (i) coffee ring, (ii) dot-like, (iii) uniform, and (iv) combined patterns. Figure 1A shows an overhead view of typical examples of the dried deposits remaining on the four substrates used with increasing hydrophobicity or decreasing wettability down each column (θ_E_ values of 59°, 77°, 107°, and 113°) and increasing surfactant concentration (0.2, 0.5, 1, 2, 5, and 10 times the CMC) across each row. Figure 1B shows a schematic classification of the deposit types observed. Figure 1C presents the average uniformity of the final dried deposits determined by percentage coverage, plotted against DTAB concentration as a ratio of the CMC, *C*/*C*_cmc_, where *C* is the DTAB concentration and *C*_cmc_ is the critical micelle concentration as previously calculated.

On the most hydrophobic substrate, PDMS, at the lowest DTAB concentration investigated, 0.2 × CMC, a combined pattern can be observed. This pattern consists of one dominant central feature, characteristic of a dot-like deposit with a thin outer ring also present, indicating a pinned contact line and the CRE at some point during the drying regime. As the DTAB concentration is increased, the coffee ring becomes the dominant feature in the patterns produced from formulations with 0.5 and 1 × CMC. The coffee ring deposits here can be distinguished from the combined pattern as although some central deposits are present, they are irregular and are distributed throughout the whole area inside the coffee ring, differing from the one primary deposit necessary for a dot-like classification. At 2 × CMC, the deposit visually appears uniform, and once the DTAB concentration is increased past this point, regular dot-like deposits with no outer ring feature are formed. Note that, for the deposits at 5 and 10 × CMC, the final deposits are notably smaller than the original solid–liquid interface of the initial droplet; thus, they are classified as dot-like despite their circular appearance, suggesting they are uniform.

For the hydrophobic and less adhesive substrate FEP, a similar pattern transition regime is observed, with combined dot-like and coffee ring patterns at lower DTAB concentrations undergoing transitions to uniform and then dot-like deposits at the higher concentrations. Again, a DTAB concentration of 2 × CMC produced the most visually uniform deposit on this substrate. On both hydrophobic substrates, uniformity determined by percentage coverage of the initial droplet contact area peaks at 97% at a DTAB concentration that is 2 times the CMC for both FEP and PDMS.

On the hydrophilic substrates, glass and PET, different pattern transition regimes were present as the surfactant concentration was increased, compared to those observed on the hydrophobic substrates. Notably, the coffee ring was the dominant pattern type for these substrates and the coffee ring features were typically more defined and pronounced. On PET, the coffee ring thickness can be seen to increase with DTAB concentration, and even when a uniform pattern is produced, at a DTAB concentration of 2 times the CMC, a slight separation can be seen between an outer coffee ring and central subfeatures. All of the patterns classified as coffee ring in this study had some form of nanoparticle deposition within the ring. While this is a common observation,^56−58^ it is worth noting that, at lower DTAB concentrations, where larger central features are present, these were differentiated from combined patterns (consisting of a dot-like central deposit with an additional coffee ring feature) due to the nature of the inner deposits. In the case of a dot-like feature, one singular central deposit is observed and the intermediate zone between the ring and the inner deposits remains clear.^11^ In the case of a coffee ring with central deposits, several larger central deposits are seen and there is more deposition visible in the intermediate zone signifying a portion of the solute not partaking in capillary flow, a common observation for suspensions with little or no surfactant acting as a dispersive agent.^23^

As with the hydrophobic substrates, a DTAB concentration that is 2 times the CMC can be seen to result in the most uniform deposits with percentage coverage peaking at 85% and 89% for glass and PET, respectively.

### 3.3 Evaporation Mode

**Figure 2:**
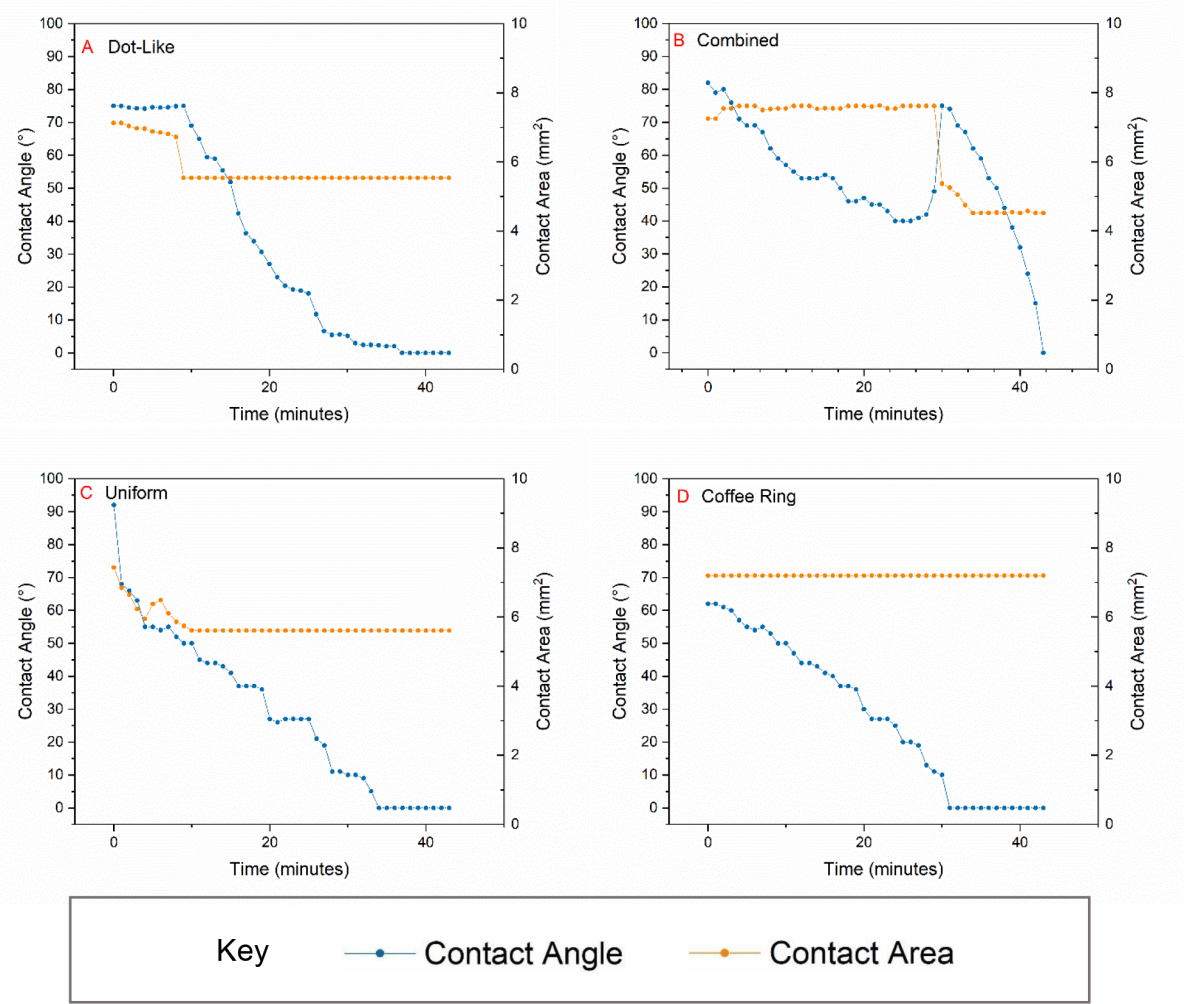
Evaporation profiles consisting of averaged data for contact area and contact angle for the entirety of the evaporation period for typical examples of specific pattern types with an initial droplet volume of 0.5 μL. (A) Data from the generation of dot-like deposits prepared from 0.5 μL droplets with a CNT loading of 3.9 mg/mL and a DTAB concentration that was 10 times the CMC deposited on PDMS. (B) Combined deposits prepared from 0.5 μL droplets with a CNT loading of 3.9 mg/mL and a DTAB concentration of 0.5 times the CMC deposited on FEP. (C) Uniform deposits prepared from 0.5 μL droplets with a CNT loading of 3.9 mg/mL and a DTAB concentration of 2 times the CMC deposited on FEP. (D) Coffee ring deposits prepared from 0.5 μL droplets with a CNT loading of 3.9 mg/mL and a DTAB concentration equivalent to the CMC deposited on PET.

Evaporation profiles were determined for each deposit type by tracking changes to the contact angle and contact area throughout the evaporation of the droplet. The data presented in Figure 2 are those of evaporated droplets with an initial volume of 0.5 μL. For dot-like deposits (Figure 2A), the initial evaporation stage takes place in CCA mode, with a slight decline in contact area signaling an unpinned contact line with relatively slow inward recession. After 9 min, a significant inward jump was made by the contact line in response to a significant depinning event in which the recession of the contact line experienced a period of significantly increased velocity. After this, the contact area remains stable as the remainder of the solvent evaporates and the contact angle decreases steadily. In Figure 2B, the profile of a combined pattern consisting of a dot-like deposit inside of an outer coffee ring is presented. After some initial spreading, the contact line and thus the contact area remain relatively constant for the first portion of the evaporation, for the first 29 min after deposition upon the substrate. Throughout this CCR phase of the evaporation, the contact angle can be seen to steadily decrease. Between 29 and 30 min, a slip event was observed as the contact line depinned and moved inward rapidly. As a result, a significant decrease in contact area and a simultaneous increase in contact angle occur. Immediately after this event, the contact area remains constant once more. Again, for the latter portion of the evaporation, the contact angle decreases steadily. The dominance of CCR in the formation of the combined deposits explains the presence of the coffee ring feature. It could also be an indicator that a strong recirculatory flow may be present. The prevalence of this pattern on low-wettability substrates suggests that hydrophobicity may contribute to the prevention of particle adsorption. Both recirculatory flow and hydrophobicity can contribute to the lack of deposition in the intermediate zone and further suggest that the particles are deposited in a central location during the final stage of evaporation.

In the formation of uniform deposits that evaporate from the formulations with a DTAB concentration of 2 × CMC (Figure 2C), a different evaporation profile is seen. Initially, from 0 to 4 min after deposition, both the contact angle and area decrease. During this period, the contact line migrates inward and simultaneously the contact angle decreases as the solvent volume decreases. A coffee ring is observed to form behind the migrating contact line. At ∼5 min, an event occurs in which the contact line becomes pinned in place. After this point, it remains pinned throughout the remainder of the evaporation process as the contact angle steadily decreases. This is observed consistently in the formation of the highly uniform deposits from samples with a DTAB concentration of 2 × the CMC, equivalent to 29 mM.

The initial phase of contact line shrinkage was observed only in the production of highly uniform deposits. For these deposits, the retraction of the contact line was coupled with contact line deposition, resulting in the formation of a ring of particles deposited over the initial solid–liquid contact area. This observation suggests the presence a capillary flow responsible for transporting the particles toward the outer edge. This accumulation of particles at the contact line could also be physically responsible for the midway pinning event.^7,59,60^

Finally, Figure 2D shows the evaporation data from a typical coffee ring deposit produced in this research. As expected, the CCR evaporation mode dominates this profile with the contact area remaining constant as evaporation proceeds.

### 3.4 Image Analysis

Image analysis was performed using ImageJ^50^ and optical microscopy images. The software was used to make deposit measurements, including radius and coffee ring thickness, and to estimate variations in particle density across the deposit, from which percentage coverage was determined to quantify deposit uniformity.

For the deposits that possessed a clear coffee ring feature at the outer edge, coffee ring thickness measurements were taken four times at 45° intervals around the perimeter of the deposit shape and averaged. These data were divided by the average deposit diameter which ranged from 515 to 2083 μm for the 0.5 μL droplets and were dependent upon both the surfactant concentration and the substrate type. The average diameter was determined for each deposit by measuring the diameter at four different points, again at 45° intervals around the deposit profile.

**Figure 3:**
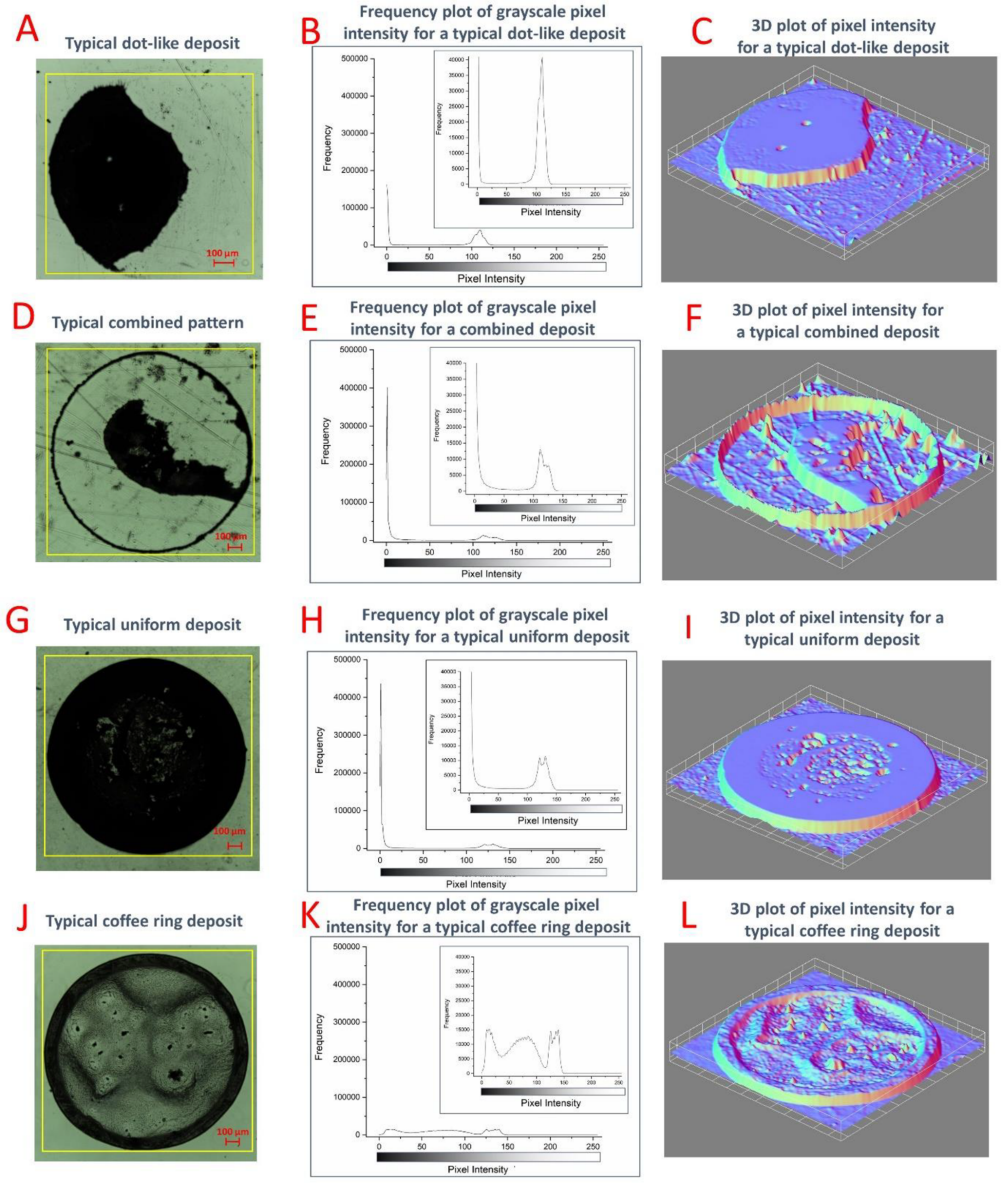
Image analysis of typical examples of the colloidal deposit patterns observed in this research. The first column shows an image of a deposit with a yellow square outlining the area selected for further analysis. The middle column presents a frequency plot of the pixel intensities present in the selected area of the image, and the final column illustrates a 3D plot of this data with pixel intensity represented by “height” so that areas of darker intensity appear higher than areas of lighter intensity. Recall that pixel intensities of 0 and 255 correspond to black and white surface colors, respectively. All data are from the deposition patterns left from the evaporation of 0.5 μL droplets with a CNT loading of 3.9 mg/mL. (A–C) Data from a droplet containing DTAB at a concentration of 0.5 times the CMC deposited on FEP. (D–F) Data from a droplet containing DTAB at a concentration equal to the CMC deposited on glass. (G–I) Data from a droplet containing DTAB at a concentration of 2 times the CMC deposited on FEP. (J–L) Data from a droplet containing DTAB at a concentration 10 times the CMC deposited on glass.

Further image analysis was undertaken, and the data are presented in Figure 3. The first column in Figure 3 shows optical microscopy images of each deposit type, for reference. The histograms in the second column plot the frequency of each pixel color against the gray scale intensity, ranging from completely black with an intensity of 0 to completely white with an intensity of 255. As the nanoparticles used are black in color, pixel intensity is used as a proxy measure of the particle density.^27,61^ The background area was included in each image analysis selection to enable comparisons between the particle free zones of the background outside of the deposit and the particle free areas within the deposit. The final column shows a 3D plot of each pattern type, in which pixel intensity is translated into a representative “height”. In these plots, the darkest, most dense regions of the deposits appear higher than areas with less coverage. Note that these plots do not indicate physical deposit height; this was analyzed via interferometry, and the results are presented in section 3.5. The 3D plots are useful for highlighting and illustrating areas of high and low nanoparticle deposition in each deposit type.

For the histogram analysis for each pattern type shown in Figure 3, a peak around 110–140 pixel intensity is present. This is the *i*_max_ value and represents the pixel intensity for the color of the substrate, free from any particle deposition.

Panels A–C of Figure 3 show data for a typical dot-like pattern, in which two dominant peaks are seen. The first, which peaks at a pixel intensity of 0 indicating complete blackness, represents the darkest areas present in the image, areas densely covered with CNT nanoparticles. The second, at a higher intensity, represents the particle free substrate. These two distinct peaks indicate areas with full or no particle coverage, indicating all suspended particles have been transported inward to the sole dot-like feature.

Figure 3D shows a histogram of pixel intensity for a typical combination deposit containing both a dot-like central feature and an outer coffee ring. Again, two peaks are present. In this instance, the absence of any other notable peaks confirms that the intermediate region in the combined deposits is in fact free from deposition. As a thin coffee ring is present, this observation quantitatively distinguishes a combined deposit from a coffee ring that may not be fully clear in the center but can possess some central features with intermediate pixel intensities as in Figure 3J. The 3D analysis in Figure 3F reveals additional elements around the large central feature, but comparisons between panels D and E of Figure 3 show such elements to predominantly consist of substrate defects and show that the remainder of the intermediate zone is relatively clear from nanoparticle deposition.

The third deposit type shown in Figure 3G–I is a uniform deposit. As with the dot-like deposit, the histogram shows just two peaks representing extremes of pixel intensity, indicating areas either densely packed or absent of particle deposition. Again, this indicates areas of either heavy or no deposition, without many intermediate intensities present. The 3D analysis does highlight a smoother area toward the deposit perimeter and an area of greater texture toward the center. This implies a coffee ring subfeature may be present within this pattern type, consistent with the events identified during the evaporation analysis of such deposits.

The histogram for the coffee ring (Figure 3K) has a much broader profile showing three interlinked peaks. The first peak representing the darkest areas is broader and at a higher pixel intensity, indicating the darkest regions are not as densely covered as those of the other deposit types analyzed. As described above, the last peak represents the substrate, but in this instance, there is a large, broad, central peak bridged between the other two. This represents the intermediate and central zones that have particle deposition ranging in density and therefore intensity. The deposition in these zones ranges from the darkest pixels observed in the coffee ring feature to the lightest pixels of the particle free substrate. This is confirmed by the 3D analysis that shows more coverage in the central region than that of the previous deposit types.

### 3.5 Profilometry

**Figure 4:**
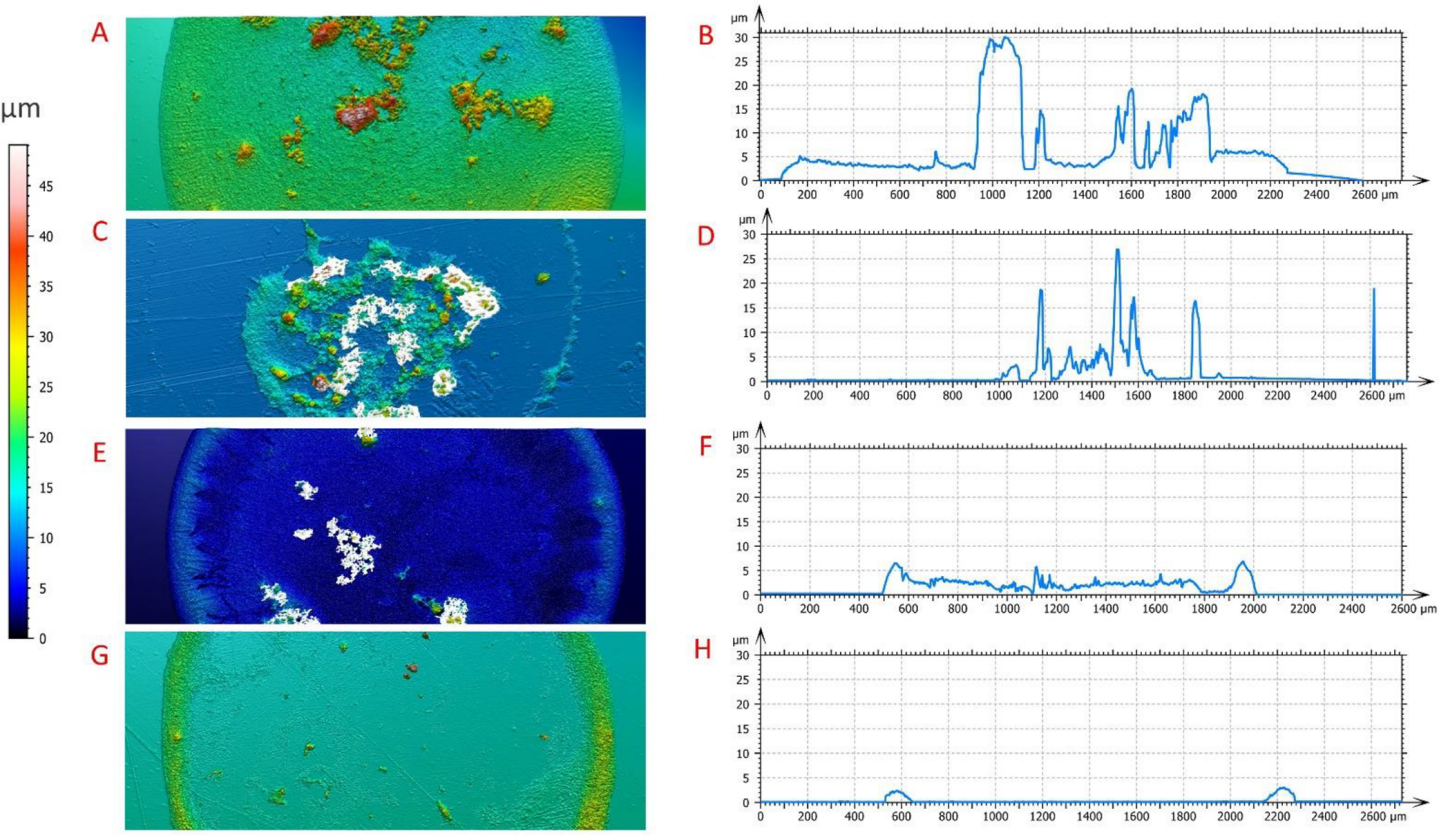
Profilometry data for typical examples of deposit types. (A and B) Data from a dot-like deposit prepared from a 0.5 μL droplet with a CNT loading of 3.9 mg/mL and a DTAB concentration of 10 times the CMC deposited on PDMS. (C and D) Data from a combined deposit prepared from a 0.5 μL droplet with a CNT loading of 3.9 mg/mL and a DTAB concentration of 0.5 times the CMC deposited on FEP. (E and F) Data from a uniform deposit prepared from a 0.5 μL droplet with a CNT loading of 3.9 mg/mL and a DTAB concentration of 2 times the CMC deposited on FEP. (G and H) Data from a coffee ring deposit prepared from a 0.5 μL droplet with a CNT loading of 3.9 mg/mL and a DTAB concentration equivalent to the CMC deposited on PET.

White light interferometry was used to analyze deposit profiles with the key findings for each deposit type highlighted in Figure 4. In addition to the profile images generated, a central cross section of each deposit type was analyzed to create a height profile, displayed in the right-hand column. Note that variations in background color are predominantly the result of variations in substrate height. For some samples deposited on PDMS, FEP, and PET, it was necessary to secure the substrate to a mount prior to analysis to level the sample, and these differences in base height were normalized for the profile plots on the right-hand side of Figure 4. For the dot-like deposit, shown in panels A and B of Figure 4, a circular relatively uniform deposit can be seen with some large agglomerates scattered throughout reaching ∼30 μm in height. This specific example of a dot-like deposit has a circular and uniform profile; however, it is worth noting that this was not reliably the case for all of the dot-like deposits, and many profiles were not as circular or symmetrical.

Panels C and D of Figure 4 represent the data from a typical combined pattern in this research, containing both a coffee ring outer feature and a central dot-like feature. In this instance, a faint and discontinuous outline of the coffee ring is visible, indicating the initial droplet radius; however, for some combined deposit types, a complete coffee ring was formed. The maximum height of the coffee ring feature in this example is 19 μm. Profile analysis confirms previous findings that the intermediate zone is indeed particle free. The large central feature varies in morphology and height, indicating a particle dumping in the latter stages of evaporation. For this deposit, the maximum height is ∼27 μm, and this peak is found in the central feature.

Panels E and F of Figure 4 illustrate the profiles of a typical uniform deposit. Despite the uniform visual appearance, profilometry indicated that a coffee ring subfeature is present. This is shown by a higher concentration of particles and therefore height at the outer edge. This subfeature was found to be present in all uniform deposits in this research regardless of the percentage coverage. The average height of the coffee ring for the uniform deposit is ∼6 μm, and the height profile shows level coverage across the central region with some larger irregular, agglomerated features still present.

Panels G and H of Figure 4 represent a coffee ring deposit with a consistent and continuous outer ring and a clear intermediate and central region. Although image analysis indicated some particle deposition in the central and intermediate zones, the profilometry analysis finds this to be very light and almost undetectable in terms of deposit height profile. The height analysis shows the coffee ring to be consistent in width and height with a peak feature height measured at only 3 μm.

### 3.6 Discussion

To understand the interplay of processes leading to the pattern formation on each substrate used, we consider each substrate in turn and analyze the pattern transition as the DTAB concentration is increased.

#### 3.6.1 Polydimethylsiloxane (PDMS)

As discussed in section 3.2, the deposition patterns observed on the hydrophobic, adhesive substrate PDMS change significantly in response to an increased surfactant concentration as they undergo a transition from a combined pattern to coffee ring (CR) to uniform and finally dot-like. Interestingly, the patterns produced on PDMS represent all of the pattern types observed in this study. For the lowest DTAB concentrations investigated, a combined pattern consisting of a coffee ring outer feature and a centralized dot is observed. It is well-known and has been reported widely that both a radially outward flow and a pinned contact line are required in the production of coffee rings;^62^ this is consistent with the findings of the evaporation analysis. As the DTAB concentration is increased to 1 × CMC, the coffee ring feature becomes more defined, suggesting an increase in the radially outward capillary flow generated inside the evaporating droplet as well as greater nanoparticle suspension. Thokcham et al.^12^ conclude that ring patterns occur on hydrophobic substrates when capillary flow is dominant inside an evaporating droplet. Conversely, in instances in which a centralized deposit is formed, it is largely the result of a dominant recirculatory Marangoni flow (MF). These findings are further backed up in a more recent study investigating plasmonically driven thermocapillary Marangoni flows to induce a coffee ring to “coffee ring with a centralized dot” pattern transition.^63^ Considering these findings and the fact that at higher DTAB concentrations dot-like deposits are produced on PDMS, one may conclude that the observed changes in pattern are the result of the competition between opposing capillary and Marangoni flows. We propose that the increase in the DTAB concentration results in a non-uniform distribution of surfactants at the liquid–air interface in the evaporating droplets, resulting in the generation of a surface tension gradient that in turn promotes a solutal MF.^64,66^

Image analysis of the uniform deposits identified a coffee ring subfeature, which indicates that even in the generation of uniform deposits, capillary flow is still present. Therefore, we conclude that a key factor in the generation of the uniform deposits at this critical concentration is that the opposing flows coexist or even cancel, resulting in the production of a thick coffee ring outer feature and a dense wide centralized feature. At concentrations above this critical concentration, MF dominates, which coupled with the hydrophobic nature of the substrate results in a transition from uniform with a coffee ring subfeature to deposits consisting of a dot-like feature only.^5,21^

#### 3.6.2 Fluoroethylene Propylene Copolymer (FEP)

On hydrophobic, non-adhesive substrate FEP, a combined to uniform to dot-like transition, similar to that observed on PDMS, occurs. The main difference is that on FEP, the coffee ring thickness does not increase with DTAB concentration, as is the case for PDMS. This is believed to be the result of the adhesive^23^ and viscoelastic properties^24^ of the PDMS that have both been found to promote contact line pinning and thus the subsequent density and thickness of coffee ring production. The absence of these characteristics on the deposits formed on FEP is believed to be responsible for the dominance of the combined pattern type at lower DTAB concentrations as increased contact line mobility promotes centralized deposition and reduces the CRE.^67^ Despite the differences between the two hydrophobic substrates, again a uniform pattern is observed at a DTAB concentration of 2 × CMC. This is believed to be the result of a complementary relationship between inward capillary and a recirculatory MF forming as the result of a surface tension gradient at the liquid–gas interface due to an increasing surfactant presence. Oh et al.^65^ identified in a recent study that increased uniformity can be achieved when capillary flow is present but becomes overwhelmed by a more dominant MF. They reported the generation of “coffee rings with islands”, with “islands” referring to centralized deposits inside of the outer coffee ring. They report that the island feature forms throughout evaporation despite the persistent presence of a weak capillary outward flow and that the manipulation of this phenomenon is an interesting avenue for investigations into increasing deposit uniformity.

#### 3.6.3 Polyethylene Terephthalate (PET) and Glass

On hydrophilic substrates PET and glass, only two pattern types (coffee ring and uniform) were observed for all of the formulations investigated.

As with the deposits on the hydrophobic substrates, the significant transition in pattern type from CR to uniform occurs at the point of the plateau of the equilibrium contact angle (see Figure S2) that occurs at a surfactant concentration equal to 2 × CMC on all substrate types. Again, deposit uniformity decreases as the DTAB concentration exceeds this point, suggesting changes to internal flow strength and particle interactions that prove to be detrimental to uniform pattern production.

#### 3.6.4 Summary

Profilometry and image analysis confirmed that for all deposits classified as uniform, a coffee ring and central deposit subfeature was present. The coffee ring thickness, determined as the ratio of deposit radius (presented in Figure S3), shows that for formulations with an observable and measurable coffee ring feature, the ring thickness increases with DTAB concentration. This is most notable for the droplets deposited on hydrophilic PET, as this substrate produced a measurable CR features for all DTAB concentrations investigated, indicative of a radially outward flow increasing in force.^11^

The persistence of the CR feature in the deposits formed from the highest DTAB concentrations (5 and 10 × CMC) on PET further supports the theory that when uniform deposits have been achieved it is due to a canceling (or perhaps complementary) relationship between outward and inward flows within the evaporating droplet. Recent work by Ren et al. determined experimentally the presence of a central disk inside an outer ring when evaporation has occurred with a pinned contact line to be the result of a dominant recirculatory MF as well as a weaker radially outward flow. Stronger MF resulted in smaller and denser central disc features, and these observations were supported by Monte Carlo simulations.^68^

In addition, we believe that altering the surfactant concentration changes the effective solvent-mediated interaction between the particles and with the substrate, and when these interactions become sufficiently attractive, particle aggregation and adhering to the substrate can also occur during the drying process.

In this research, uniform deposits were achieved at a specific DTAB concentration across a range of hydrophilic and hydrophobic substrates used. The percentage uniformity of the deposits was found to decrease on either side of a critical DTAB concentration, suggesting that particle–particle interactions are more influential than particle–substate interactions, as the DTAB concentration was the only variable on each substrate series. This could be an indication that the solute concentrations used are in excess of what can be adsorbed at the free interfaces rendering any adsorption at the liquid–air interface negligible in comparison to the effect of the behavior of the particles within the droplet bulk.^69^

## 4 Conclusions

In summary, we have identified a specific concentration, 26.3 mM, of cationic surfactant DTAB that can mitigate the coffee ring effect in mwCNT-laden aqueous droplets and significantly increase deposit uniformity over a range of substrates with varying properties. This specific concentration is equivalent to 2 times the experimentally determined CMC, and uniformity was found to decrease on either side of this concentration on all substrates used in this study. The plateau in surface tension value as a result of an increasing DTAB concentration occurs at ∼2 times the CMC for both the surfactant stock solutions and the mwCNT-laden formulations. We present evidence in the Supporting Information illustrating this. First, the Supporting Information presents a plot of DTAB concentration versus sample surface tension. From this and Young’s equation below, we can infer that as the liquid–gas interfacial surface tension (*γ*_lg_) decreases, so too does the equilibrium contact angle (θ)
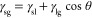
where *γ*_sl_ and *γ*_sg_ are the solid–liquid and solid–gas interfacial tensions, respectively. We can therefore conclude that surface tension decreases in the same manner even with the addition of the nanoparticles. Additionally, in the Supporting Information, we also present plots of the formulation equilibrium contact angle and the coffee ring thickness, both plotted as a function of the DTAB concentration on each substrate. These taken together confirm that the wetting and interfacial adsorption behavior of the mwCNT-laden and mwCNT free surfactant suspensions is similar, confirming comparable micelle formation behavior.

Evaporation analysis indicates that a self-pinning event after some initial contact line recession was present in the generation of uniform deposits. This event was also responsible for an outer ring subfeature, identified by profilometry and image analysis, present in all uniform deposits in this research. This self-pinning event is likely the result of a physical buildup of particles at the contact line^7^ rather than an accumulation of particles forming along the liquid–gas interface and altering the interfacial properties^19^ due to the lack of such events in the evaporation of formulations with a higher solute concentration. This observation applied to droplets on both hydrophilic and hydrophobic substrates, further implying that this mode of drying is the result of physical interactions between particles within the droplet, rather than external parameters or particle–substrate interactions.

These results are of particular interest, because undesired self-assembly in evaporating complex droplets often creates unwanted effects and many existing methods for mitigating these effects are either costly or too complex. Some recent examples include applying a magnetic field in the evaporation of an iron oxide solution on PDMS,^70^ heating to high temperatures (between 130 and 250 °C) for a variety of inkjet inks,^71^ irradiating with infrared laser beams for calcium sulfate suspensions on hydrophobic PDMS and hydrophilic polydopamine substrates,^57^ and exposure to air flow for polytetrafluoroethylene on both glass and silicon wafer substrates.^72^ Although these methods offer effective CRE suppression, they are complicated to use and generally require the utilization of costly specialized equipment. A much simpler, and therefore more appealing, approach is to include additives with the colloidal liquid that suppress the CRE during drying.

Much research on evaporative dynamics and self-assembly of complex droplets focuses on spherical nanoparticles, namely polystyrene, while particles with more complex shapes such as those with high aspect ratios have been much less studied. CNT nanoparticles like those investigated in this research have applications in a variety of fields from tissue engineering^73^ to energy storage.^74^ One expanding field in which they show great promise is that of printed electronics. However, as droplet deposition-based techniques such as inkjet printing rely on the evaporation of particle-laden droplets, they are highly susceptible to the negative effects of undesired self-assembly, such as the CRE. Our work provides a new cost-effective and simple method for producing uniform deposits from complex droplets of carbon nanotubes on a range of hydrophilic, hydrophobic, adhesive, and non-adhesive substrates.

## Supporting Information Available

Experimental determination of CMC (Figure S1), equilibrium contact angle θ_E_ as a function of DTAB concentration for each formulation used on each substrate (Figure S2), and coffee ring thickness (Figure S3) (PDF)
