# Supplementary material for: Surfactant Control of Coffee Ring Formation in Carbon Nanotube Suspensions
Source: Langmuir. 2023 Jan 6;39(3):929–41. doi: 10.1021/acs.langmuir.2c01691 (PMC9878724; doi:10.1021/acs.langmuir.2c01691)

Surfactant Control of Coffee Ring Formation in Carbon Nanotube Suspensions

N. S. Howard* (A) (N.S.Howard@lboro.ac.uk)

A. J. Archer (B,C) (A.J.Archer@lboro.ac.uk)

D. N. Sibley (B,C) (D.N.Sibley@lboro.ac.uk)

D. J. Southee (D) (D.J.Southee@lboro.ac.uk)

K. G. U. Wijayantha (A,E) (U.Wijayantha@lboro.ac.uk)

(A) Department of Chemistry, Loughborough University, Loughborough, LE11 3TU, UK
(B) Department of Mathematical Sciences, Loughborough University, Loughborough, LE11 3TU, UK
(C) Interdisciplinary Centre for Mathematical Modelling, Loughborough University, Loughborough, LE11 3TU, UK
(D) School of Design and Creative Arts, Loughborough University, Loughborough, LE11 3TU, UK
(E) Centre for Renewable Energy Systems, Cranfield University, Cranfield, Bedford, MK43 0AL, UK

Corresponding Author: Naomi S. Howard

Email: N.S.Howard@lboro.ac.uk

Phone Number: +44 (0)1509 223600

2.10 Physical General Chemistry Lab, Department of Chemistry, Sir David Davis Building, Loughborough University, LE11 3TU

Figure S1

Experimental determination of CMC: Plot of the surface tension as a function of DTAB concentration obtained using and optical tensiometer in pendant drop mode.

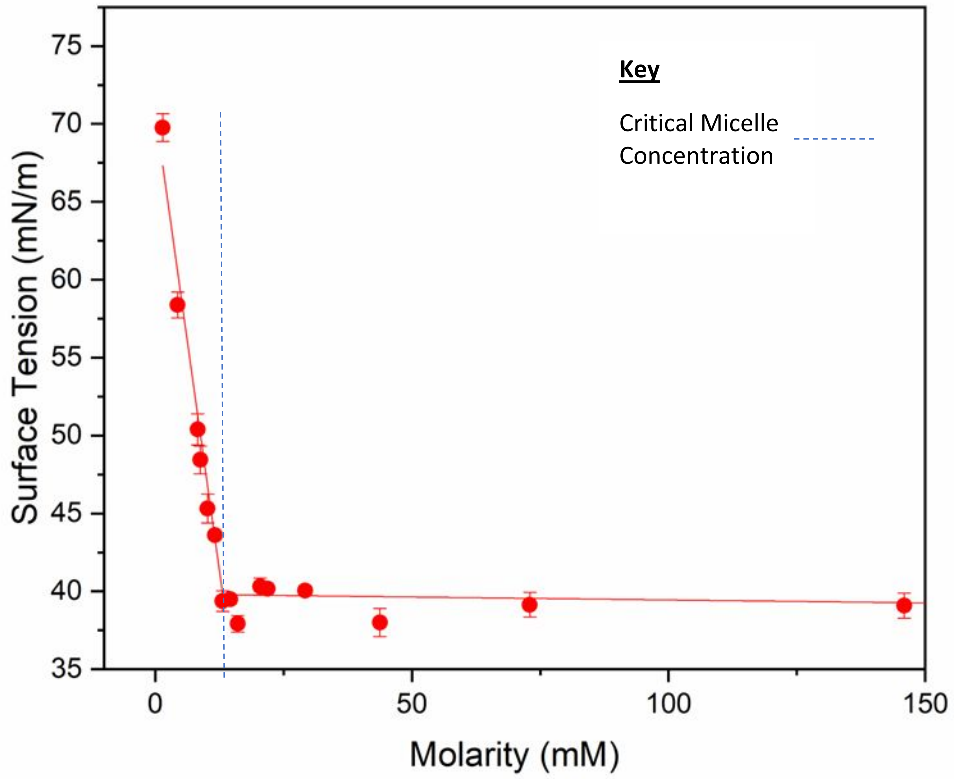

Figure S2

Equilibrium contact angle θ_E as a function of DTAB concentration for each formulation used on each substrate. For all contact angles measured standard deviation was less than 2°.

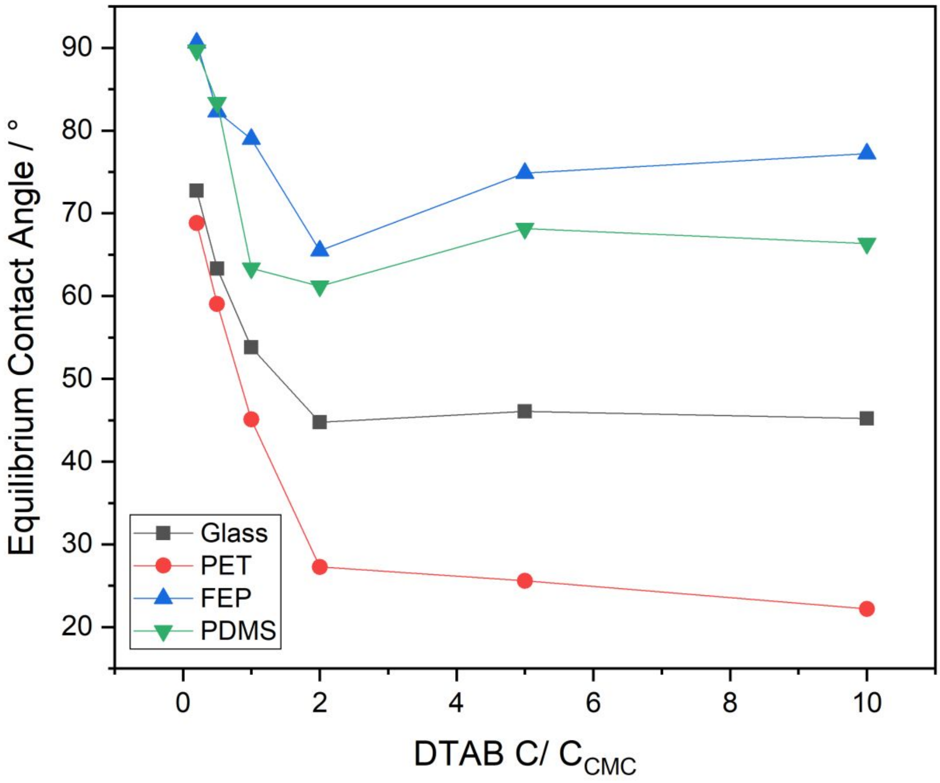

Figure S3

Coffee Ring Thickness: Diameter ratio as a function of DTAB concentration.

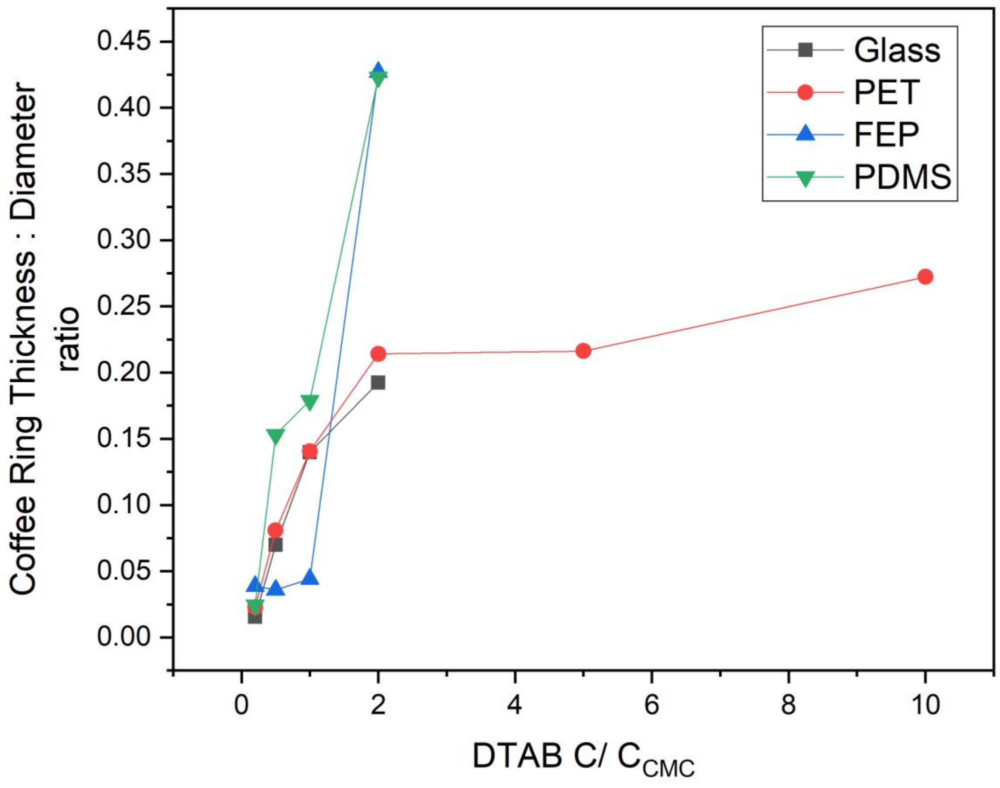

Supplement: Supplementary file 1 — la2c01691_si_001.pdf [file la2c01691_si_001.pdf]
